# Awareness in Primary School Teachers regarding Traumatic Dental Injuries in Children and Their Emergency Management: A Survey in South Jaipur

**DOI:** 10.5005/jp-journals-10005-1335

**Published:** 2016-04-22

**Authors:** Mitakshara Nirwan, Ather Ahmed Syed, Shefali Chaturvedi, Puneet Goenka, Swati Sharma

**Affiliations:** 1 Postgraduate Student, Department of Pediatric and Preventive Dentistry, Mahatma Gandhi Dental College and Hospital, Jaipur, Rajasthan, India; 2 Assistant Professor, Department of Pedodontics, Jazan University, Jazan Kingdom of Saudi Arabia; 3 Reader, Department of Pediatric and Preventive Dentistry, Mahatma Gandhi Dental College and Hospital, Jaipur, Rajasthan, India; 4 Associate Professor, Department of Pediatric and Preventive Dentistry, Mahatma Gandhi Dental College and Hospital, Jaipur, Rajasthan, India; 5 Associate Professor, Department of Pedodontics, Buddha Institute of Dental Sciences, Patna, Bihar, India

**Keywords:** Dental trauma, Emergency management, Teachers.

## Abstract

**Introduction:** Trauma to primary and permanent teeth and their supporting structures is one of the most common dental problems seen in children. The prognosis of traumatized teeth depends on timely attention with prompt and appropriate treatment, which often relies on knowledge of the teachers who may be present at the place of accidents. Thus, the aim of this study was to evaluate via a questionnaire the knowledge level of primary school teachers in South Jaipur regarding dental trauma.

**Design:** Questionnaire survey.

**Materials and methods:** A self-designed questionnaire was administered to 300 primary school teachers from 20 randomly selected private and semi-aided schools of South Jaipur.

**Results:** A total of 278 teachers responded to the survey. The collected data were subjected to statistical analysis. It was found that most of the respondents had accepted poor knowledge regarding dental trauma, with a mean knowledge of 10.56 ± 2.58.

**Conclusion:** This study highlighted inadequate knowledge regarding emergency management of traumatic dental injuries, and teachers felt the need for training in the management of dental trauma as part of their training program.

**How to cite this article:** Nirwan M, Syed AA, Chaturvedi S, Goenka P, Sharma S. Awareness in Primary School Teachers regarding Traumatic Dental Injuries in Children and Their Emergency Management: A Survey in South Jaipur. Int J Clin Pediatr Dent 2016;9(1):62-66.

## INTRODUCTION

Traumatic dental injuries (TDIs) are one of the main causes for morbidity and mortality of teeth, especially anterior teeth. Traumatic dental injuries are highly prevalent from infancy to adolescence.^1^ Epidemiological studies have revealed that children from 8-12 years often suffer from dental injuries.^2^ Dental trauma may vary from minor tooth fracture to extensive dentoalveolar damage involving supporting structures and tooth displacement or avulsion.^3^ Its treatment is complicated and can be quite expensive. In addition, follow-up visits may be necessary for many years which adds to the total expenditure on the patients’ side. Boys usually report more TDIs than girls because of their active participation in sports and games. The peak incidence in boys is 2―1 and 9-10 years and in girls is 2-3 years. The teeth most commonly involved are maxillary central incisor (37%), mandibular central incisor (18%), mandibular lateral incisor (6%) and maxillary lateral incisor (3%).^4^

Oral factors (increased overjet with protrusion), environmental determinants (material deprivation) and human behavior (risk-taking children, children being bullied, emotionally stressful conditions, attention-deficit hyperactivity disorder and violence) were found to increase the risk for TDIs.^5,6^

While small enamel loss or cracks represent minor TDIs and do not require immediate attention, severe TDIs that involve both hard and soft tissues require prompt emergency treatment. Such an action would ensure pain control, restoration of function/esthetics and prevention of social or psychological consequences.^7^ However, technical knowledge and clinical experience are essential to establish an accurate diagnosis and provide a rational treatment. For some types of traumatic injuries, prognosis is highly dependent upon proper emergency management immediately after the traumatic incident as well as timely attention by a professional.^8^ Previous reports indicate that the majority of TDIs occur at school^9,10^ and highlight the importance of trained/ experienced school staff, who are most often required to respond initially to the traumatic incident. Thus, there is an utmost need to increase the knowledge of teachers vis-á-vis recommended protocol for emergency management of TDI. Educational initiatives planned to inform teachers can positively influence their knowledge and attitudes toward emergency management of dental trauma, consequently leading to a more favorable prognosis.^11^ However, recent reports have shown that teachers have poor knowledge about dental trauma and its emergency management.^12-17^

This study aimed to evaluate via a questionnaire the knowledge level of primary school teachers in South Jaipur, Rajasthan, regarding dental trauma and to enlighten them about the subject.

The objective of this study was to assess the awareness and identify the factors associated with teachers’ knowledge related to dental trauma and to describe them the importance of immediate attention and prompt treatment in cases of TDIs.

## MATERIALS AND METHODS

The total number of primary school teachers in the South Jaipur region was estimated to be 1,020. For this cross-sectional survey, taking 95% confidence level, confidence interval (margin of error) of 5 and population variance (P) of 50%, the minimum calculated sample size required was 278. The study was executed by administering a self-designed questionnaire to 300 primary school teachers from 20 randomly selected private and semi-aided schools of South Jaipur using cluster random sampling method. The questionnaire was divided into three parts: Part I, demographic details, such as, age, gender, education level and previous experience of dental trauma were recorded, Part II, knowledge-based questions were designed and last in Part III, attitude of teachers toward education of dental trauma management was assessed. The respondents were asked to tick the most appropriate answer in order to assess their awareness and knowledge regarding emergency management of TDI. An initial pilot survey was done with the questionnaire being administered to 30 schoolteachers, and subsequently Cronbach’s alpha was calculated, which yielded a value of 0.79 validating this self-designed questionnaire.

## RESULTS

**Fig. 1:**
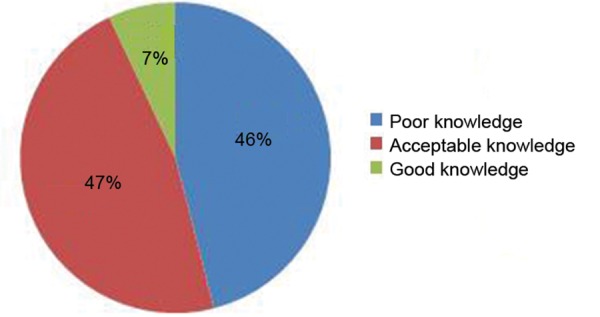
Overall knowledge distribution

Out of 300 a total of 280 schoolteachers returned the questionnaire, thereby achieving a response rate of 93.3%. Results expressed as frequency distribution tables were analyzed using Statistical Packages for the Social Sciences (SPSS) version 16.0. Basic demographic distribution of the subjects is illustrated in Table 1. The overall mean age of the respondents was 35.01 ± 8.12 years. Most of the respondents had dental trauma knowledge ranging from acceptable to poor (130 and 132 respectively), with a mean knowledge of 10.56 ± 2.58. The overall knowledge distribution is illustrated in Figure 1.

**Table 1:** **Table 1:** Basic demographic distribution of the subjects

		*Attributes*		*Value n (%)*	
Age		20-30 years		91 (32.4)	
		31-40 years		118 (42)	
		41-50 years		49 (17.4)	
		>51 years		22 (7.8)	
Gender		Females		205 (73.2)	
		Males		75 (26.8)	
Years of experience		< 5 years		83 (29.6)	
		> 5 years		197 (70.4)	

Chi-square test was used for significant associations of knowledge distribution with age, gender and years of teaching experience. The association of age with knowledge was statistically significant, showing a p value of <0.02 (Table 2), whereas the association of gender and years of experience with knowledge was statistically nonsignificant, showing a p value of 0.027 and 0.73 respectively (Tables 3 and 4).

**Table 2:** **Table 2:** Association of age with knowledge

				*Knowledge*							
*Age (years)*		*Total*		*Poor*		*Acceptable*		*Good*		*p-value*	
20-30		91		46 (50.5%)		45		0			
31-40		118		44 (37.2%)		64		10		0.002	
41-50		49		24 (48.9%)		19		6		
>51		22		16 (72.7%)		4		2			

**Table 3:** **Table 3:** Association of gender with knowledge

		*Knowledge*							
*Gender*		*Poor*		*Acceptable*		*Good*		*p-value*	
Male		29		40		6		0.27	
Female		101		92		12			

**Table 4:** **Table 4:** Association of years of experience with knowledge

		*Knowledge*							
*Years of experience*		*Poor*		*Acceptable*		*Good*		*p-value*	
Less than 5		38		41		4		0.73	
More than 5		92		91		14			

Responses for self-assessment and knowledge questions are illustrated in Tables 5A and B. It was observed that only 12.5% had received previous first-aid training about dental injuries. About 74.6% of the respondents claimed that they were able to distinguish deciduous from permanent teeth, whereas only 33.2% knew that maxillary front tooth present in an 8-year-old child is a permanent tooth. Also less as 8.6% of respondents possessed the knowledge of how to correctly manage fractured teeth. Only 31% of the respondents knew how to manage displaced teeth; 56.8% of the respondents said that they would try to locate an avulsed tooth, whereas 55% correctly answered that avulsed permanent teeth should be replanted. Very few respondents (31%) were able to correctly answer the measures to clean an avulsed tooth; 36.4% felt that immediate dentist’s opinion is needed in case of avulsed teeth. Moreover, only 20.4% of the respondents could correctly identify one of the appropriate mediums for storing an avulsed tooth.

**Table 5A:** **Table 5A** Self-assessment questions

*Questions*		*Frequency*		*Percentage*	
Have you ever encountered a dental trauma in a child ?					
a. Yes		89		31.8	
b. No		150		53.6	
c. Do not remember		41		14.6	
Do you supervise the children during sport activities?					
a. Yes		200		71.4	
b. No		31		11.1	
c. Sometimes		49		17.5	
Can you do emergency management in case of dental trauma?					
a. Yes		70		25	
b. No		167		59.6	
c. Do not know		43		15.4	
Did you have first-aid training of the dental trauma?					
a. Yes		35		12.5	
b. No		240		85.7	
c. Do not know		5		1.8	
Does the school have first aid safety kit?					
a. Yes		70		25.0	
b. No		167		59.6	
c. Do not know		43		15.4	
Are you satisfied with your knowledge on “the management of dental trauma™?					
a. Yes		60		21.4	
b. No		220		78.6	
Do you think it is important to have an educational program in “management of dental trauma™?					
a. Yes		280		100	
b. No		0		0	
Would you like to attend any educational program on “management of dental trauma™?					
a. Yes		280		100	
b. No		0		0	

**Table 5B:** **Table 5B** Knowledge-based questions

*Questions*		*Correct* *response*		*Incorrect* *response*	
What would you do if your student falls down while running and breaks his upper front tooth?		4		276	
If the tooth is broken, is the broken part important according to you?		127		153	
What would you do if your student fell and had a trauma to his teeth and is having slight mobility in his upper right front tooth?		56		224	
Your student’s upper left tooth is dislocated. What would you do in this case?		180		100	
Your 8-year-old student got hit on his mouth, his upper front tooth is found to be missing/fractured. The damaged front teeth likely to be a permanent or a primary tooth?		93		187	
Will you try to find the knocked down tooth?		159		121	
The ideal time for replacing the tooth?		132		204	
The correct time to approach dentist for opinion in case of knocked down tooth?		102		178	
If the knocked down tooth has become dirty what would you do?		88		192	
How would you keep the tooth till you reach the dentist?		59		227	

## DISCUSSION

Traumatic dental injuries are highly prevalent from infancy to adolescence and often occur in schools, causing alterations in the child’s facial development, psychological changes in behavior besides other complications.^18,19^ Teachers are likely to be in contact with the students soon after an episode of TDI. It is their knowledge of emergency procedures that is crucial in ensuring a better prognosis of the clinical treatment.^20^ Almost all the earlier studies have also observed that majority of the respondents had experienced TDI in children at schools and considered the knowledge regarding dental trauma management essential.^21^ Hence, this study aimed to investigate the awareness levels in primary school teachers regarding TDIs and related emergency management.

The association of knowledge with age was found to be statistically significant, suggesting that age of the respondents had influence on knowledge regarding TDI. The association of gender and years of teaching experience with knowledge regarding TDI was statistically insignificant.

The study revealed that although 31.8% of the respondents had encountered a dental trauma in a child, and most common type of trauma encountered was fracture of the crown followed by avulsion of the teeth respectively, alarmingly most of the teachers had little or no information regarding emergency management of TDI. Also most of the teachers (85.7%) have not received any first-aid training for management of dental trauma. Similar findings were also reported in a study done by Bhandary and Shetty,^22^ in which 75.3% teachers had not received training for managing dental trauma.

Most of the teachers were inadequately equipped to handle fractured teeth as only 1.4% thought it was important to locate fractured segments, while majority of them (71%) opted to stop the bleeding and do nothing else. These results were in agreement with a study done by Arikan and Sonmez,^8^ where a total of 7.6% respondents said that they would try to find the broken tooth piece. Also only 45.3% teachers realized the importance of the broken part of the tooth.

Around 47.8% of the participants were not able to recognize whether the damaged front tooth in an 8-year-old child is primary or permanent, which is very important in determining the emergency treatment and prognosis of the tooth. However, the present survey found that most of the teachers (74.6%) were confident enough to distinguish between deciduous and permanent teeth: These findings are similar to a study done by Bhandary and Shetty^22^ where 72.4% of teachers were confidently able to distinguish between deciduous and permanent teeth.

Significant number of respondents (55%) were not conscious of the fact that the knocked out permanent tooth can be replanted within a limited period of time after it has been cleaned and disinfected. Teachers were also not aware about the critical role of time in replacing an avulsed tooth (52.9%). These findings were in line with the writings of Prasanna et al,^23^ who concluded that as little as 23% of teachers knew the management of severe tooth injuries, such as tooth avulsion. Most of the earlier studies also showed that the teachers advocated transferring the dentally injured patients with avulsed tooth to a dentist. They, however, did not agree in taking any active role in managing avulsed tooth trauma.

The present study showcased that among the respondents, knowledge vis-a-vis cleaning and maintenance of the avulsed tooth in a storage medium was inadequate, as most of them believed that tap water was a favorable medium to maintain/wash the avulsed teeth. These findings were similar to the study by Hashim.^21^ Despite years of research showing that cell membranes will be destroyed if stored in normal saline, not as much number of teachers (18.9%) thought that a tooth could be stored in such a medium. However, on a positive note, all of the respondents agreed that educational programs to manage TDI are necessary. This is in line with the findings of Hashim^21^ and Bhandary and Shetty,^22^ who had noted that a great majority of the respondents were not satisfied with the knowledge on the ‘management of dental trauma’ and most of them expressed a desire for further information.

The effective initial management or emergency management reduces the risk and severity of the condition. An understanding of traumatic injuries will help the teachers to have a keener eye on injuries and fractures among school children.

The present study, thus, revealed that optimum and essential knowledge about emergency management of dental trauma of primary teachers in South Jaipur is grossly insufficient. It can be summarized by stating that all the schoolteachers should have the basic knowledge to recognize and handle oral emergencies, especially regarding conservation of avulsed teeth to prevent its consequences in the child’s future.

Due to the limitation of this study being restricted to only one region of Jaipur city, the data presented may not fully represent the knowledge levels of primary school teachers of the entire city. Therefore, a city- and perhaps state-wide investigation could be conducted to shed valuable light on this important subject matter.

## CONCLUSION

This study highlighted the substantial lack of knowledge regarding emergency management of TDIs. Thus, strategies to improve the teachers’ knowledge about dental trauma must be planned along with the inclusion of this topic in the teachers’ curricular training in a continuous manner. The dentist’s, especially pediatric dentist’s, role should be to create awareness among persons who are directly associated with the upbringing of children, regarding the importance of prompt treatment in cases of dental trauma. This can be achieved by conducting camps and educating them through posters, advertisements and audiovisual aids.
